# The description of protein internal motions aids selection of ligand binding poses by the INPHARMA method

**DOI:** 10.1007/s10858-012-9662-1

**Published:** 2012-09-22

**Authors:** Benjamin Stauch, Julien Orts, Teresa Carlomagno

**Affiliations:** 1 Structural and Computational Biology Unit, European Molecular Biology Laboratory (EMBL), Heidelberg, Germany; 2 Present Address: European Bioinformatics Institute (EBI), Hinxton, UK; 3 Present Address: Eidgenössische Technische Hochschule (ETH), Zurich, Switzerland

**Keywords:** INPHARMA, Protein dynamics, Ligand binding, Order parameters

## Abstract

Protein internal motions influence observables of NMR experiments. The effect of internal motions occurring at the sub-nanosecond timescale can be described by NMR order parameters. Here, we report that the use of order parameters derived from Molecular Dynamics (MD) simulations of two *holo*-structures of Protein Kinase A increase the discrimination power of INPHARMA, an NMR based methodology that selects docked ligand orientations by maximizing the correlation of back-calculated to experimental data. By including internal motion in the back-calculation of the INPHARMA transfer, we obtain a more realistic description of the system, which better represents the experimental data. Furthermore, we propose a set of generic order parameters, derived from MD simulations of globular proteins, which can be used in the back-calculation of INPHARMA NOEs for any protein–ligand complex, thus by-passing the need of obtaining system-specific order parameters for new protein–ligand complexes.

**Electronic supplementary material:**

The online version of this article (doi:10.1007/s10858-012-9662-1) contains supplementary material, which is available to authorized users.

## Introduction

Protein surfaces are not static but plastic boundaries, interacting with and adapting to ligands. Besides steric and electrostatic interactions, dynamic features of proteins and protein–ligand interactions have been shown to be functionally relevant (Karplus and Kuriyan 2005; Kay et al. 1998). Protein dynamics can be probed experimentally and computationally (Hub and de Groot 2009; Kohn et al. 2010), with NMR spectroscopy standing out as an especially well-suited experimental tool to study the dynamics of complexes (Mittermaier and Kay 2006) in a close-to-native liquid environment.

NMR spectroscopy is particularly powerful in the investigation of transient protein–ligand complexes (Carlomagno 2005). Ligand binding epitopes can be mapped using STD techniques (Jayalakshmi and Krishna 2002; Mayer and Meyer 2001), protein residues contacting the ligand can be identified using chemical shift perturbation experiments (McCoy and Wyss 2002), and transferred-NOEs or transferred-CCR (cross-correlated relaxation) rates allow for the determination of the bioactive conformation of the ligand (Blommers et al. 1999; Ni 1994; Carlomagno et al. 1999).

Recently, we have developed the INPHARMA method (Orts et al. 2008; Sanchez-Pedregal et al. 2005) to determine the relative binding mode of two competitive, transiently bound ligands. INPHARMA relies on interligand, protein-mediated, transferred-NOE signals between two ligands L_A_ and L_B_, binding competitively and weakly to a receptor T. The efficiency of the INPHARMA transfer at each ligand site depends on the relative binding mode of the ligands to the protein; the transfer is generally more efficient between two protons of the two ligands that are close to the same protein protons in the binding pocket (Supplementary Figure S1). Because of this dependency, a quantitative analysis of the INPHARMA NOEs allows for the determination of the relative binding mode of L_A_ and L_B_ to the target protein. In favorable cases, the absolute orientation of the ligands within the protein binding pocket can be determined as well from INPHARMA data (Orts et al. 2008). In agreement with standard structural based drug-design workflows, the INPHARMA data are used to select the correct binding modes from a pool of pairs of complex structures generated by molecular docking. The agreement between experimental and back-calculated INPHARMA data for each complexes pair is used as selection criterion (Reese et al. 2007).

For Protein Kinase A (PKA) and two competitive ligands L_A_ and L_B_, the INPHARMA NOEs allowed selection of the correct ligand binding poses from a pool of pairs of PKA/L_A_ and PKA/L_B_ structures representing combinations of very different orientations of the ligands (Orts et al. 2008, 2012). A high correlation coefficient is found between experimental and back-calculated INPHARMA NOEs for the complexes’ pair representing the correct ligands binding poses, that is the crystal structures of the PKA/L_A_ and PKA/L_B_ complexes. In this favorable case, the INPHARMA data allowed a clear selection not only of the relative, but also of the absolute binding mode of both L_A_ and L_B_.

Despite the high correlation coefficient (R = 0.82) of the experimental INPHARMA NOEs with the data back-calculated for the correct structures of the PKA/L_A_ and PKA/L_B_ complexes, the experimental data were consistently lower than the theoretical ones (slope of the linear fit = 0.33), indicating overestimation of the magnetization transfer efficiency (Orts et al. 2008, 2009). To explain this effect, we suggested the influence of protein internal motion on the INPHARMA NOEs, as order parameters smaller than 1 would reduce the efficiency of the magnetization transfer. Since the interligand NOEs observed in an INPHARMA experiment are mediated by protein protons through spin-diffusion, their value depends on protein internal motions. This is in contrast to intraligand transferred-NOEs, which are dominated by the direct dipolar–dipolar interaction between protons of the ligand and are only mildly affected by the protein protons.

In this work, we set out to include NMR order parameters in the INPHARMA calculations. To demonstrate the usefulness of order parameters in improving the quality of the fit of experimental to theoretical data and in increasing the selective power of the INPHARMA NOEs, we first estimate a set of order parameters for the PKA/L_A_ and PKA/L_B_ complexes from Molecular Dynamics simulations; next, we modify the implementation of the full relaxation matrix approach (Nilges et al. 1991), used to back-calculate the INPHARMA data, to allow for incorporation of order parameters in the spectral density function. Lastly, we introduce a set of generic order parameters to be used in INPHARMA calculations, thus by-passing the need of performing MD simulations on each protein/ligand complex of interest; we show that even the use of generic, non-tailored order parameters increases the discrimination power of the INPHARMA method.

## Theory

### Protein internal motions

Dipolar cross-relaxation rates σ_kl_^NOE^ determine magnetization transfer between spins *k* and *l* through space and depend on the spectral density functions (Ernst et al. 1987):

1 $$ J_{\text{kl}} (\omega ) = \int\limits_{ - \infty }^{\infty } {C_{\text{kl}} (t)\exp ( - i\omega t){\text{d}}t} $$

which are the Fourier transforms of the dipolar correlation functions:

2 $$ C_{\text{kl}} (t) = 4\pi \left\langle {\frac{{Y_{20} \left( {\theta_{\text{kl}}^{{1{\text{ab}}}} (t_{0} + t)} \right)Y_{20}^{*} \left( {\theta_{\text{kl}}^{{1{\text{ab}}}} (t_{0} )} \right)}}{{r_{\text{kl}}^{3} (t_{0} + t)r_{\text{kl}}^{3} (t_{0} )}}} \right\rangle $$

with $\theta_{\text{kl}}^{{1{\text{ab}}}}$ being the angle between the inter-nuclear vector *r*_kl_ and the external magnetic field, *Y*_2*m*_ being the rank 2 spherical harmonics of order m and the angled brackets denoting a Boltzmann ensemble average. Assuming that the overall tumbling motion of the molecule is much slower than fast internal motion, the two kinds of motions can be treated independently of each other (Wallach 1967): for isotropic diffusional tumbling, the correlation function of the overall motion is an exponential $C^{\text{tumbling}} (t) = e^{{ - |t|/\tau_{c} }}$ with τ_*c*_ being the correlation time of the molecule. The contribution of the internal motions to the dipolar correlation function has the form:

3 $$ C_{\text{kl}}^{\text{internal}} (t) = \frac{4\pi }{5}\sum\limits_{m = - 2}^{2} {\left\langle {\frac{{Y_{2m} \left( {\theta_{\text{kl}}^{\text{mol}} (t_{0} + t),\phi_{\text{kl}}^{\text{mol}} (t_{0} + t)} \right)Y_{2m}^{*} \left( {\theta_{\text{kl}}^{\text{mol}} (t_{0} ),\phi_{\text{kl}}^{\text{mol}} (t_{0} )} \right)}}{{r_{\text{kl}}^{3} (t_{0} + t)r_{\text{kl}}^{3} (t_{0} )}}} \right\rangle } $$

with *r*_kl_^3^, θ_kl_^mol^, and ϕ_kl_^mol^ being the spherical coordinates in a molecular fixed frame.

For *t* → ∞ the internal correlation function *C* ^internal^(*t*) assumes a plateau value S^2^, which is called the NMR order parameter (Lipari and Szabo 1982).

The dipolar spectral density functions can then be rewritten as (Brueschweiler et al. 1992):

4 $$ J_{\text{kl}} (\omega ) = \left\langle {\frac{1}{{r_{kl}^{ 6} }}} \right\rangle S_{kl}^{2} \frac{{2\tau_{c} }}{{1 + \omega^{2} \tau_{c}^{2} }} + \left\langle {\frac{1}{{r_{kl}^{ 6} }}} \right\rangle (1 - S_{kl}^{2} )\frac{{2\tau_{tot} }}{{1 + \omega^{2} \tau_{tot}^{2} }} $$

where

5 $$ S_{\text{kl}}^{2} = \frac{4\pi }{5}\left\langle {r_{\text{kl}}^{ - 6} } \right\rangle^{ - 1} \sum\limits_{m = - 2}^{2} {\left| {\left\langle {\frac{{Y_{2m} (\theta_{\text{kl}}^{\text{mol}} ,\phi_{\text{kl}}^{\text{mol}} )}}{{r_{\text{kl}}^{3} }}} \right\rangle } \right|^{2} } $$

and

6 $$ \frac{1}{{\tau_{tot} }} = \frac{1}{{\tau_{c} }} + \frac{1}{{\tau_{kl} }} $$

and τ_kl_ being the internal correlation time. The second term of Eq. (4) can be neglected if τ_kl_ ≪ τ_c_.

Assuming that the angular and the radial fluctuations are uncorrelated, the order parameter of Eq. (5) can be factorized as:

7 $$ S_{\text{kl}}^{2} \approx S_{{{\text{r}},{\text{kl}}}}^{2} \cdot S_{{\Upomega ,{\text{kl}}}}^{2} $$

where

8 $$ S_{\text{r,kl}}^{2} = {{\left\langle {r_{\text{kl}}^{ - 3} } \right\rangle^{2} } \mathord{\left/ {\vphantom {{\left\langle {r_{\text{kl}}^{ - 3} } \right\rangle^{2} } {\left\langle {r_{\text{kl}}^{ - 6} } \right\rangle }}} \right. \kern-\nulldelimiterspace} {\left\langle {r_{\text{kl}}^{ - 6} } \right\rangle }} $$

and

9 $$ S_{{\Upomega ,{\text{kl}}}}^{2} = \frac{4\pi }{5}\sum\limits_{m = - 2}^{2} {\left| {\left\langle {Y_{2m} (\theta_{\text{kl}}^{\text{mol}} ,\phi_{\text{kl}}^{\text{mol}} )} \right\rangle } \right|^{2} } $$

are the radial and angular contributions, respectively. As previously observed (Brueschweiler et al. 1992), for PKA this was found to be true to a good approximation (data not shown).

## Results

### INPHARMA calculations using order parameters

In previous work (Orts et al. 2008) conducted on PKA in complex with two ligands L_A_ and L_B_ (Supplementary Figure S2), we had reported a high correlation coefficient for the linear fit between experimental INPHARMA NOEs and INPHARMA NOEs calculated from crystal structure distances in the complexes PKA/L_A_ and PKA/L_B_ (Pearson correlation coefficient R = 0.82). In this work, internal motions of both the protein and the ligands had been neglected (S_kl_^2^ = 1 for all pairs of protons (k, l)). Despite the high correlation coefficient of the linear fit, the slope of only 0.33 indicated the systematic overestimation of the magnetization transfer (Orts et al. 2008) by a factor of ~3.

In order to explain the deviation of the slope of the linear fit from 1, we explored the impact of including internal motions in the INPHARMA calculations. Protein internal motions are expected to have an impact on the values of interligand INPHARMA NOEs, as these NOEs are mediated by the protons of the protein via spin diffusion. Unlike transferred-NOEs, which have been shown to depend mostly on direct interactions between protons of the ligand, INPHARMA NOEs strictly depend on the interaction of the ligand(s) with the protein protons.

**Fig. 1:**
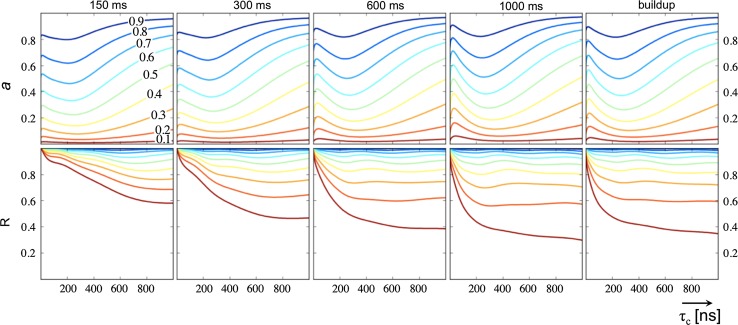
Plots for the parameters of linear fits of INPHARMA NOEs calculated for the PKA/L_A_ and PKA/L_B_ complexes with uniform order parameters S^2^ < 1 versus reference INPHARMA NOEs calculated for the rigid case with S^2^ = 1. Slopes (*upper panel*) and Pearson correlation coefficients (*lower panel*) of best fit lines are shown in dependence of complex size (x axis, τ_c_ = 1–1,000 ns) and order parameter S^2^ (*contour lines*) for different mixing times (*left* to *right*) and for the full build-up consisting of combined data from all four mixing times. *Contour lines* at values [0.1;0.9] are shown* color-coded* (*red* to *green* to *blue*). All combinations of INPHARMA NOEs between the groups of protons of Fig. S2 have been calculated; data are normalized to diagonal peak intensities in a NOESY spectrum at 150 ms mixing time

In order to provide a general understanding of the influence of internal motions on INPHARMA NOEs, we simulated the effect of uniform order parameters S^2^ < 1 of different size for both the protein and the ligands in both the free and bound states (Fig. 1). The data were calculated for the system consisting of the PKA/L_A_ and PKA/L_B_ complexes, for which the correlation time is varied artificially between 0 and 1,000 ns to simulate the effect of receptor size. For a medium-sized protein (τ_c_ = 15–20 ns), the intensities of the INPHARMA NOEs are very sensitive to internal motion; on the other hand, for large receptors lower order parameters are tolerated before observing a considerable effect on the slope of the fit. This is due to a compensatory effect in large receptors, where S^2^ < 1 reduces the efficiency of the INPHARMA transfer at the protein–ligand interface (as it does for smaller receptors) but at the same time reduces the loss of ligand magnetization in the protein core due to spin-diffusion. In the absence of internal motions, the loss of ligand magnetization due to spin-diffusion in the protein core is more prominent for large receptors than for small ones; consequently, larger receptors benefit more from slowing down this process. Our results emphasize the importance of considering internal motions for small and medium-sized systems. At the same time, the discriminatory power of the INPHARMA calculations proves remarkably robust with respect to variations of the order parameters for both small and large receptors, as correlation coefficients stay high over a wide range of order parameter values, especially when using full build-up information.

Next we asked the question about the dynamics of which group of proton pairs has the strongest effect on the magnitude of magnetization transfer. We systematically varied the order parameters for the inter-molecular and intra-molecular NOEs for a fixed receptor size (τ_c_ = 17 ns) and monitored the intensity of the INPHARMA NOEs (data not shown). As expected, the reduction of the order parameters for the inter-molecular NOEs has the largest effect and reduces the efficiency of the protein-mediated magnetization transfer between the two ligands. This reduction can be compensated by the presence of S^2^ < 1 for the intra-protein NOEs, which, as explained before, reduces the loss of magnetization in the protein core. Intra-ligand order parameters S^2^ < 1 contribute the least.

The calculations of Fig. 1 and Supplementary Figure S3 predict that a uniform order parameter S^2^ < 1 of about 0.5 is necessary to cause a three-fold reduction of the INPHARMA NOEs for the PKA system. To verify whether a more realistic representation of the protein and ligand dynamics would be able to explain the observed slope of 0.33 in the fit between the experimental and the back-calculated INPHARMA NOEs for the PKA/L_A_ and PKA/L_B_ complexes, we obtained estimation of order parameters in the complexes from trajectories of Molecular Dynamics (MD) simulations (Brueschweiler et al. 1992). We performed 30 ns MD simulations of the PKA/L_A_ and PKA/L_B_ complexes and extracted a set of order parameters for all proton pairs within 10 Å of the ligand binding pocket that have less than 6 Å mutual inter-nuclear distance. Due to these cutoffs, proton pairs that are not included in the calculations would either have mutual distances beyond NOE detection, or be too distant from the binding pocket for spin-diffusion mediated magnetization transfer. To remove the overall tumbling motion of the molecule, each frame was superimposed to the crystal structure as common reference frame. The order parameter was factorized in the radial and angular part according to Eq. (7), which resulted to be a good approximation for our system (correlation coefficient R > 0.996 between S_kl_^2^ and product S_r,kl_^2^ · S_Ω,kl_^2^). The order parameters extracted from the MD simulations have an average of 0.62 ± 0.22, in good agreement with order parameters derived from MD simulations in another study (Schneider et al. 1999). Spectral density functions containing the first term of Eq. (4) were used in the full relaxation matrix to calculate the INPHARMA NOEs for the PKA/L_A_ and PKA/L_B_ complexes with the correct ligands orientations.

To our disappointment, the incorporation of internal motions in the INPHARMA calculations considerably deteriorated the quality of the fit between experimental and back-calculated data, yielding R = 0.66. However, the slope increased to 0.71, suggesting that internal motions can indeed explain the over-estimation of the INPHARMA NOEs in back-calculation performed for rigid complexes.

The decrease in the value of the theoretical INPHARMA NOEs of more than two-fold upon inclusion of internal fluctuations indicates that the internal motions primarily affecting the INPHARMA NOEs are of angular nature. A strong effect of radial fluctuations would in fact increase the rate of the NOE-transfer, according to $\left\langle {r_{kl}^{ - 3} } \right\rangle^{2} \geq \left\langle r \right\rangle^{ - 6} ,$ and therefore result in a decrease of the slope of the correlation between experimental and theoretical data.

The poor fit obtained with the order parameters and distance averaging from the MD runs indicates that Molecular Dynamics is not able to reproduce the motional features of the complexes at a high level of accuracy. This is in line with the notion that obtaining accurate quantitative predictions of the NMR relaxation parameters from MD simulations is a challenging task (Trbovic et al. 2008; Markwick et al. 2008; Case 2002), despite recent improvements in force-field parameterization (Showalter et al. 2007). In general, MD simulations are able to reproduce order parameters for backbone NH and methyl group CH bond vectors measured by NMR (Showalter and Brueschweiler 2007; Ming and Brueschweiler 2004), which contain only the angular part of Eq. (7). Also in our hands, quantitative estimation of backbone NH and methyl group order parameters works reasonably well on a benchmark set of 4 globular proteins (see below). It is therefore reasonable to assume that in this case the discrepancy between the experimental and the back-calculated data can be mainly attributed to the failure of MD simulations to reproduce the distance average $\left\langle {r_{kl}^{ - 3} } \right\rangle^{2}$ or $\left\langle {r_{\text{kl}}^{ - 6} } \right\rangle .$ A similar conclusion is reported also in (Vogeli et al. 2009); in our case the situation is aggravated by the intermolecular ligand–protein distances, which are even more challenging to reproduce theoretically due to the much worse definition of ligand force fields with respect to protein force fields.

To verify the ability of the MD simulation to reproduce the correct distance distribution, we compared the average distances $\left\langle {r_{kl} } \right\rangle$ from the MD simulations with the distances extracted from the crystal structure, which can be considered a good approximation of the average state in solution and the most accurate distance information available. This comparison reveals that the correlation between *r*_*kl,cryst*_ and $\left\langle {r_{kl} } \right\rangle$ is of poor quality for interproton distances below 6 Å (R = 0.65), with the MD-derived distances being consistently larger than the statistic distances in the crystal structures. This is in agreement with a recent study on perdeuterated ubiquitin (Vogeli et al. 2009), which shows that inaccuracies in order parameter estimations from MD simulations can be attributed to distance effects and MD derived distances exhibit a poor correlation to NMR distances derived from cross-relaxation measurements. In our case, if only the intermolecular distances between the protein and the ligand are considered, the quality of the correlation between *r*_*kl,cryst*_ and $\left\langle {r_{kl} } \right\rangle$ drops even further (R = 0.18 for the PKA/L_A_ complex), which underlines the inability of MD simulations to correctly reproduce the motions of the ligand in the binding pocket.

Inaccuracies in the MD simulations, especially in the short distance range, which dominates the average, are expected to have a large effect on $\left\langle {r_{\text{kl}}^{ - 6} } \right\rangle ,$ while the effect on $S_{{{\text{r}},{\text{kl}}}}^{2} = {{\left\langle {r_{\text{kl}}^{ - 3} } \right\rangle^{2} } \mathord{\left/ {\vphantom {{\left\langle {r_{\text{kl}}^{ - 3} } \right\rangle^{2} } {\left\langle {r_{\text{kl}}^{ - 6} } \right\rangle }}} \right. \kern-\nulldelimiterspace} {\left\langle {r_{\text{kl}}^{ - 6} } \right\rangle }}$ is expected to be less (for example, 10 % error on the internuclear distances translates to 10 % error on the radial order parameter *S*_r,kl_^2^, but 25 % error on $\left\langle {r_{\text{kl}}^{ - 6} } \right\rangle ,$ as observed in numerical simulations). Therefore, while the MD-derived averaged distances $\left\langle {r_{\text{kl}}^{ - 6} } \right\rangle$ are substantially wrong, the MD-derived radial order parameters $S_{{{\text{r}},{\text{kl}}}}^{2} = {{\left\langle {r_{\text{kl}}^{ - 3} } \right\rangle^{2} } \mathord{\left/ {\vphantom {{\left\langle {r_{\text{kl}}^{ - 3} } \right\rangle^{2} } {\left\langle {r_{\text{kl}}^{ - 6} } \right\rangle }}} \right. \kern-\nulldelimiterspace} {\left\langle {r_{\text{kl}}^{ - 6} } \right\rangle }}$ are expected to be closer to the correct ones. We therefore decided to use the S_r,kl_^2^ order parameters derived from MD simulations, while turning to alternatives for the estimation of $\left\langle {r_{\text{kl}}^{ - 6} } \right\rangle .$

In the absence of an accurate estimation for $\left\langle {r_{\text{kl}}^{ - 6} } \right\rangle$ in Eq (4) from MD simulations, we attempted substituting $\left\langle {r_{\text{kl}}^{ - 6} } \right\rangle$ with $r_{kl,cryst}^{ - 6} .$ This choice was made following previous work, which reported on a good correspondence between the effective distances extracted from NOE data and distances from crystal structures. In particular, (Vogeli et al. 2009) showed that for backbone NH interproton distances up to 5 Å in perdeuterated ubiquitin, the crystal structure distances are generally within 5 % of effective averaged distances extracted from NOESY NMR experiments. This result suggests that for most distances $r_{kl,cryst}^{ - 6}$ might be a good surrogate of $\left\langle {r_{\text{kl}}^{ - 6} } \right\rangle ,$ as expected for internal motions of moderate amplitude.

**Fig. 2:**
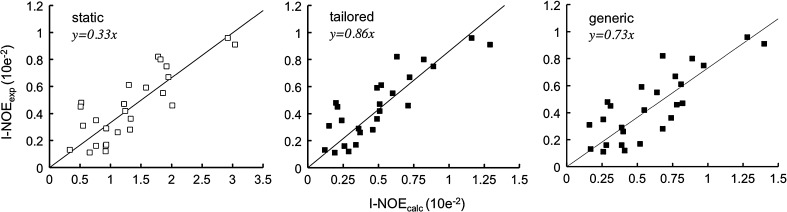
Linear regression of experimental INPHARMA-NOEs (I-NOE_exp_) at mixing times 300, 450, 600, and 750 ms versus simulated data (I-NOE_calc_) ignoring (*left panel*) and considering (*central* and *right panels*) internal motions. In the *central panel*, tailored order parameters, derived from MD-simulations for the PKA, L_A_, L_B_ system, are used; in the *right panel* generic order parameters are used. INPHARMA cross-peak intensities are normalized to diagonal peak intensities of L_A_ in a NOESY spectrum at mixing time of 150 ms. Best-fit lines (y = *a*x, *black*) are plotted after performing a linear regression (*left*, R = 0.82, *a* = 0.33; *centre*, R = 0.86, *a* = 0.86, *right*, R = 0.81, *a* = 0.73)

Using $r_{kl,cryst}^{ - 6}$ as a surrogate for $\left\langle {r_{\text{kl}}^{ - 6} } \right\rangle$we are able to improve the correlation of the back-calculated INPHARMA NOEs to the experimental data (R = 0.86 vs. R = 0.82 in the static case) (Fig. 2); the slope of the linear fit reaches 0.86, which indicates that internal motions on a fast time-scale are responsible for most of the over-estimation of the INPHARMA NOEs in back-calculations using static complexes. Interestingly, setting a uniform order parameter of 0.62 (equal to the average of all order parameters extracted by the MD runs) would only result in a slope of 0.6, indicating that our set of order parameters captures specific characteristics of the interaction.

In any case, in a routine application of INPHARMA, $\left\langle {r_{\text{kl}}^{ - 6} } \right\rangle$ would not be available from MD simulations for any protein and ligand binding pose of interest, due to the expensiveness of the calculation. A feasible approach could consist in back-calculating the INPHARMA NOEs using static distances from complex structural models in combination with generic order parameters that can be applied to any protein. In view of these limitations, the fact that the experimental data for the PKA/L_A_ and PKA/L_B_ system can be best reproduced by using the crystal structure static distances and the order parameters extracted by MD simulations is encouraging. In the next paragraph we explore the possibility of defining generic order parameters that can be used for any protein/ligand complex in the INPHARMA back-calculations.

At this point it should be noted that the usage of an incorrect correlation time for the free ligands can also alter the slope of the correlation between the back-calculated and the experimental INPHARMA NOEs (Zheng and Post 1993). Therefore it is essential to optimize the parameters of the system, such as the correlation time of the complex and the correlation time of the free ligands, by fitting the intraligand transferred-NOEs before calculating the INPHARMA NOEs.

### Generic order parameters

MD simulations are computationally costly and it is unfeasible to run them for every application of INPHARMA. In addition, current force fields are designed for proteins, while simulations including ligands require manual adjustment and extension of the force field. To by-pass the demanding task of running MD simulations, it would be desirable to obtain a set of order parameters that can be easily transferred between systems.

To generate such *generic* order parameters, containing both angular and radial contributions according to Eq. (5), we follow the following approach: from MD simulations of different globular proteins, we derive order parameters for pairs of non-exchanging protons; we decompose our order parameters into contributions of individual protons (*vide infra*) and assign them to different chemical groups; last, we propose to use group-wise averaged values as *generic* order parameters that represent the motional behavior of equivalent groups in any system of interest.

In general, order parameters *S*_kl_^2^ are defined for pairs of nuclei (k, l). To derive generic order parameters for each proton type in a protein, the first required step is the decomposition of S_kl_^2^ in proton-specific contributions *S*_k_ and *S*_l_ such that ${\text{S}}_{\text{kl}}^{2} \approx S_{\text{k}} \cdot S_{\text{l}} .$ To find the optimal values S_k_ for all protons k, we minimize the *l* ^2^-norm (Euclidean norm) of the difference ${\mathbf{A}} - {\mathbf{x}}^{\text{T}} {\mathbf{x}},$ with A_kl_ = S_kl_^2^, to obtain a vector **x** with x_k_ = S_k_. In other words, we decompose the pair-wise order parameter S_kl_^2^ into contributions from the single protons k and l; by minimizing the target function for all protons at the same time, all available dynamic information about the protein is considered, i.e. for a given nucleus k, S_k_ contains information about all S_kl_^2^ for k ≠ l.

Non-exchanging protons are classified according to the heavy atom they are covalently bound to, yielding 9 groups: C-α, CH_3_, CH_2_-β, CH_2_-γ, CH_2_-δ, CH_2_-ε, CH_2_-proline, CH_1_, and aromatic protons. Each of the groups is split in two sub-groups, depending on whether the proton k belonging to that group is involved in a dipolar interaction with a proton l belonging to the same or to another residue. On average, we find that every proton has 18 neighbors within the defined distance cutoff (5 Å), 6 of which belong to the same residue, and the remaining 12 are in other residues (11 in non-neighboring residues). The average *S*-factor, calculated over the S_k_ for all protons inside one of the eighteen defined chemical classes, represents the *generic* *S*-factor for that class.

To transfer this information and assign expected motional behavior to an unknown system, we assign to each proton k of the system an atom-type, according to the grouping scheme described above, along with the corresponding *generic* *S*-factor S_k_. The generic order parameter S_kl_^2^ defined for the dipolar interaction between k and any other proton l is obtained by multiplying the respective *S*-factors S_k_ and S_l_.

We obtain the set of expected *S*-factors from 25 ns long MD simulations of 4 globular proteins, for which a high-resolution crystal structure is available in the Protein Data Bank (Berman et al. 2000) (human ubiquitin, PDB identifier 1ubq (Vijay-Kumar et al. 1987); human FYN tyrosine kinase SH3 domain, PDB identifier 1shf (Noble et al. 1993); the murine adipocyte lipid binding protein, PDB identifier 1lib (Xu et al. 1993); fibronectin type III domain from human tenascin, PDB identifier 1ten (Leahy et al. 1992). For the internal correlation function C(t)^internal^ to be considered to converge to S^2^, we require it to be constant within a range around the plateau value for an extended time. An extensive description of how order parameters are extracted is given in the Methods section. The dynamics of each of these proteins has been investigated experimentally by NMR spectroscopy (Best et al. 2004; Constantine et al. 1998; Lee et al. 1999; Mittermaier et al. 2003) and can thus be used to benchmark our ability to estimate order parameters from MD simulations. For human ubiquitin, experimental order parameters can be reproduced well by our MD simulation (R = 0.87, root mean square error, RMSE = 0.15 for methyl groups and R = 0.75, RMSE = 0.07 for the backbone); for the human FYN tyrosine kinase SH3 domain, methyl axis order parameters are in excellent agreement with experiments (R = 0.76, RMSE = 0.13), while backbone order parameters are not available; for both the murine adipocyte lipid binding protein and the fibronectin type III domain from human tenascin, methyl axis order parameters are well reproduced (R = 0.84, RMSE = 0.14 and R = 0.71, RMSE = 0.21, respectively), while the reproduction of backbone order parameters is less accurate (R = 0.53, RMSE = 0.14 and R = 0.62, RMSE = 0.05, respectively). For the murine adipocyte lipid binding proteins, the lower quality of the fit of backbone order parameters can be explained by the fact that the dynamics studies were conducted on the human protein, while the crystal structure of the human protein was not available at the time when we performed the simulations. The human and the murine proteins differ in 11 residues, which can explain the differences in the dynamics. Similarly, for the fibronectin type III domain from human tenascin, the dynamic studies were performed on a 2 amino acids longer construct (aa 1–92), while the crystal structure is on a truncated construct (aa 1–90). Hamill et al. report that the C-terminal extension has a stabilizing effect on the protein and alters its dynamics. Interestingly our studies can detect differences in the dynamic behavior of different constructs (Hamill et al. 1998).

Encouraged by the apparent good quality of the MD simulations in reproducing (angular) fluctuations, we used the sets of theoretical order parameters from the MD simulations of all four proteins to derive generalized order parameters. After decomposition of the order parameters S^2^ to obtain *S*-factors, these *S*-factors are averaged for each proton class for all four proteins (Table 1). To judge the quality of the decomposition, we compute linear correlation coefficients between S_kl_^2^ and the product S_k_ · S_l_ for all pairs of nuclei (k, l). For the four test systems, we obtain average correlation coefficients R of 0.979 (intra-residue, RMSE 0.045–0.055) and 0.935 (inter-residue, RMSE 0.076–0.085), respectively.

**Table 1:** *S*-factors for “inter-” and “intra-residue” proton pairs averaged over the four globular proteins (Table S1)

	Inter-residue (SD)	Intra-residue (SD)	N (inter/intra)
C-α	0.96 (0.01)	0.93 (0.03)	372/297
CH_3_	0.62 (0.03)	0.56 (0.03)	611/587
CH_2_-β	0.87 (0.02)	0.81 (0.04)	366/307
CH_2_-γ	0.78 (0.04)	0.73 (0.04)	154/160
CH_2_-δ	0.69 (0.04)	0.60 (0.02)	52/67
CH_2_-ε	0.44 (0.04)	0.39 (0.05)	35/48
CH_2_-proline	0.82 (0.03)	0.77 (0.04)	62/64
CH_1_	0.92 (0.03)	0.79 (0.06)	98/99
Aromatic	0.78 (0.02)	0.88 (0.03)	121/124

Both angular and radial fluctuations are contained in these values

*N* number of protons used to derive the corresponding *S*-factor; *SD* standard deviation of each value

The values we obtain reflect the expected dynamic behavior of a protein: C-α protons are largely static, while methyl group rotation results in low *S*-factors for CH_3_ protons; for the different CH_2_ subgroups, an increasing mobility (as reflected in decreasing *S*-factor) can be appreciated when moving away from the main chain (from β to ε). It should be noted that the angular contribution to the *S*-factor dominates the intra-residue values (that is, the order parameter between closer protons), while the radial contribution becomes more important for the inter-residue values (that is the order parameter between protons at longer distance) (data not shown).

### Performance of generic order parameters in INPHARMA calculations

Next, we used the set of generic order parameters in the INPHARMA calculations for the PKA system. Protein hydrogens of PKA were assigned to the chemical groups as defined above; the ligand hydrogens were assigned to equivalent groups, as if they belonged to the protein, i.e. the “aromatic” or “CH_2_” group. This is an approximation; however, the lack of reliable force fields for organic ligands precludes a reliable calculation of ligand order parameters from MD simulations, leaving no other choice than using this approximation. *S*-factors of individual protons were re-multiplied to retrieve generic order parameters, and used in the INPHARMA calculations. Similar to what observed with the set of PKA-specific order parameters, generic order parameters result in an increased slope of the best fit line of 0.73; the correlation coefficient R of 0.81 is only slightly worse than that obtained with PKA-specific order parameters and similar to that obtained for the rigid case (Fig. 2). This result confirms the usefulness of the generic order parameter for the INPHARMA back-calculations.

### Validation of calculated order parameters

**Fig. 3:**
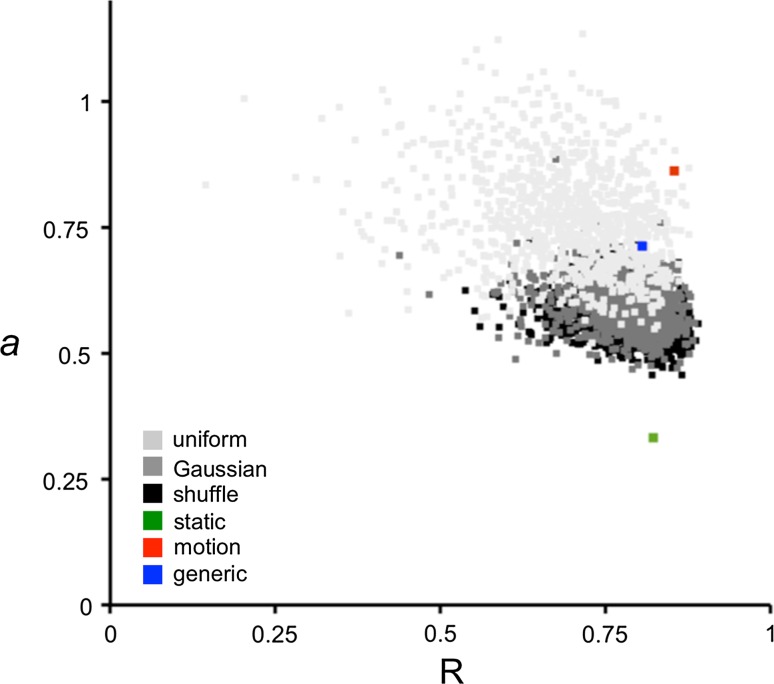
Effect of randomization of the order parameters set. The INPHARMA NOEs, which are back-calculated using different sets of (randomized) order parameters are linearly fit to the experimental data. The values of the slopes *a* of the respective best-fit line (y axis) is plotted against the Pearson correlation coefficient R of the fit (x axis). Each dot represents a set of order parameters. The *color code* is as follows: *red*, the tailored order parameters extracted from MD simulation; *blue*, the generic set of order parameters; *green*, the *rigid case*; *black*, sets of order parameters randomized by shuffling proton pairs and order parameters; *dark gray*, sets of order parameters drawn from a Gaussian distribution with mean 0.62 and SD 0.22 to resemble the order parameter dataset extracted from MD simulation; *light gray*, a set of order parameters drawn uniformly from [0;1]

To further validate our set of order parameters, we investigate its performance with respect to randomization (Fig. 3). We create different sets of random order parameters and evaluate how well these sets reproduce the experimental data in the INPHARMA calculations in comparison to the performance of the original set. Each test is iterated 1,000 times, each time using a different random set of order parameters. We perform three tests: (1) we shuffle the original set, i.e. to each pair of nuclei, we randomly assign the order parameter originally derived for another pair of nuclei; (2) we assign random order parameters drawn from a Gaussian distribution centered at 0.62 and with standard deviation 0.22; (3) we assign random order parameters drawn uniformly from [0;1]. Figure 3 shows that both the shuffled and the Gaussian set of order parameters cluster in the same region, while the uniform dataset samples a wider range of quality space. Our sets of tailored and generic order parameters, however, dominate most solutions according to the Pareto criterion of multidimensional optimization. This demonstrates that the set of order parameters extracted from the MD simulation is appropriate to describe differential dynamics in the protein.

### Impact of order parameters on the discriminatory power of INPHARMA

The generic order parameters can be used in INPHARMA calculations of any complex of interest. A relevant question is whether the representation of internal motions through the generic order parameters can improve the selection of the correct ligand binding modes, for example by providing an improved clear-cut discrimination in the linear fit of the experimental data to the correct or wrong binding poses.

**Fig. 4:**
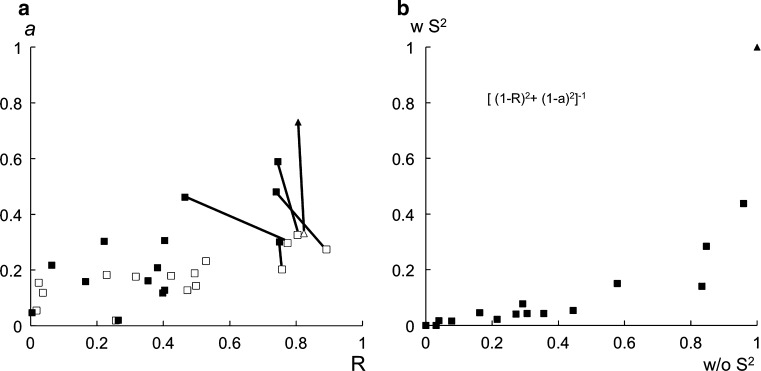
Comparison of the selectivity of INPHARMA, when including (*solid symbols*) or excluding (*empty symbols*) internal motions, for the PKA/L_A_ and PKA/L_B_ complexes represented by a test set of four binding poses per ligand (yielding 16 ligands combinations). The correct pair of ligands binding poses, as seen in the crystal structures of PKA/L_A_ and PKA/L_B_ (PDB IDs 3dne and 3dnd, respectively), is indicated as* triangle*; other, incorrect, solutions as *squares*. **a** Slope of best fit line *a* plotted against Pearson correlation coefficient R for rigid and motional models. Equivalent solutions with R > 0.70 for the rigid model are connected by *black lines*. **b** Combined quality factor of motional against rigid model; Pearson correlation coefficients R and slopes of best fit line *a* are combined according to the formula [*m*(1 − R)^2^ + *n*(1 − *a*)^2^]^−1^ with *m* = *n* = 1, and resulting values are normalized to the interval [0;1]

To answer this question, we use the experimental data for the complexes PKA/L_A_ and PKA/L_B_ to select among the 16 pairs of complexes analyzed in (Orts et al. 2008). These complex pairs result from the combination of four different binding modes of L_A_ and four different binding modes of L_B_, which all differ from each other by 180º rotations around three orthogonal axes. Figure 4a shows the correlation coefficient and the slope of the linear fits of the experimental data versus the back-calculated data for all 16 pairs with (filled symbols) and without (empty symbols) including the generic order parameters in the INPHARMA calculations. If internal motions are ignored, four models show a correlation coefficient higher than 0.8 and therefore pass the INPHARMA selection. As explained in (Orts et al. 2008), further discrimination between the binding poses is obtained by additional criteria, such as the systematic deviation of INPHARMA peaks stemming from different structural moieties of the ligands and the semi-quantitative use of further weak INPHARMA peaks. However, when using the generic order parameters to describe the internal motions, a much better discrimination of the binding modes is achieved. Both the high correlation coefficient and the slope point to the PKA/L_A_ and PKA/L_B_ complexes pair indicated with a triangle as the one best reproducing the experimental data; these complexes correspond to the correct binding pose for both ligands, as seen in the crystal structures.

In alternative to evaluating the correlation coefficient and the slope separately, a composite quality factor of the type [*m*(1 − R)^2^ + *n*(1 − *a*)^2^]^−1^ can be applied to select the pairs of binding modes that is best in agreement with the experimental data. Figure 4b shows this composite quality factor for the sixteen combinations of PKA/L_A_ and PKA/L_B_ models, both considering (y axis) and ignoring (x axis) internal motions. The better discrimination between poses achieved when including the description of internal motions through the generic order parameters in the INPHARMA back-calculations is striking (Fig. 4b). As a note of caution, we point out that the impact of the generic order parameters on INPHARMA calculations has been tested for one experimental system only (PKA/L_A_ and PKA/L_B_ complexes). A more extensive validation through experimental data for other complex systems, including proteins of different sizes, is in progress in our laboratory. In general, we expect a larger impact of order parameters in the discrimination potential of INPHARMA NOEs for proteins of smaller size (Fig. 1).

## Conclusions

In this work, we present an extension of the INPHARMA method by incorporating protein plasticity in the calculations. By improving the realism of the underlying physical model, i.e. by incorporating NMR order parameters representing protein internal motions into the spectral density function, we achieve an improvement of the correlation coefficient between simulated and experimental data (0.86 as opposed to 0.82 for the rigid case) and of the slope of the linear regression line (0.86 as opposed to 0.33 for the rigid case). This confirms that the systematic over-estimation of magnetization transfer observed when treating the protein as rigid is largely accounted for by the use of order parameters. Importantly, we suggest generic order parameters that can be used for any experimental system irrespective of atomic coordinates. For the PKA system, we find that the use of generic order parameters to represent internal motions improves the efficacy of INPHARMA in selecting between different ligands binding poses.

## Materials and methods

### MD simulations and force field parameterization

Proteins and protein–ligand complexes were simulated using NAMD 2.6 (Phillips et al. 2005) and the CHARMM22 force field (MacKerell et al. 1998) in a periodic cubic box of explicit TIP3 water with a side length of the maximum internuclear distance of the respective protein atoms (~45 Å for 1lib, ~50 Å for 1shf, ~47 Å for 1ten, and ~45 Å for 1ubq), plus a padding of 25 Å to avoid mutual interaction of the protein images. Crystal structure coordinates, with hydrogens added with REDUCE (Word et al. 1999), were used as starting points. After 10^4^ steps of initial energy minimization, systems were heated stepwise from 0 to 298 K with a temperature increment of 3 K per 1 ps, followed by an equilibration phase of 5 ns and the production runs. The time step of the integrator was set to 2.0 fs and a Langevin thermostat was applied. Force field parameters of ligands (equilibrium bond lengths, angles, dihedral angles, and non-bonded interactions) were assigned analogous to known compounds as described previously (Vanommeslaeghe et al. 2010). Analogous substructures were extracted from thiazole, thiophene, histidine, indole, and aminopyridine moieties, and corresponding parameters assigned to unknown ligand parameters. After MD simulation, each frame of the trajectory was superimposed to the crystal structure conformation, minimizing protein heavy atom RMSD. Water coordinates were deleted and one snapshot per 1 ps was subjected to further analysis.

### Extraction of order parameters

Distance-dependent NMR order parameters (Lipari and Szabo 1982) were calculated directly from the MD trajectories utilizing VMD (Humphrey et al. 1996) according to Eq 5 (Brueschweiler et al. 1992). Purely radial and purely angular order parameters were calculated as in Eqs. (8) and (9). (Brueschweiler et al. 1992).

### Criterion for convergence of correlation functions

The existence of an order parameter S_kl_^2^ requires the internal correlation function *C*_*kl*_(*t*) to converge to a plateau value. After removing overall tumbling motion by superimposing each snapshot to the initial structure as a reference, normalized internal correlation functions were computed directly from the MD trajectory as:

10 $$ C_{kl} (t_{i} ) = c_{1} \frac{1}{N - i}\sum\nolimits_{j = 1}^{N - i} {\frac{{P_{2} \left( {\theta_{kl}^{lab} (t_{j} ) \cdot \theta_{kl}^{lab} (t_{i + j} )} \right)}}{{r_{kl}^{3} (t_{j} ) \cdot r_{kl}^{3} (t_{i + j} )}}} $$

with C_kl_(t) containing both angular and radial fluctuations and P_2_(x) = ½(3x^2 ^− 1) the second Legendre polynomial, $\theta_{kl}^{lab} /r_{kl}(t_{j} )$ the unit vector orientation/distance between nuclei k and l at time t_j_, respectively, N the finite length of the MD trajectory and $c_{{_{1} }}^{ - 1} = \frac{1}{N - i}\sum\nolimits_{j = 1}^{N - i} {r_{kl}^{ - 6} } (t_{j}) .$ It should be noted that the choice of the normalization constant is arbitrary and was set to correspond to the definition of the order parameter (Eq. 5) for convenience.

For rapid internal motions, C decays rapidly to a plateau value S^2^ with a characteristic internal correlation time. As MD trajectories are of finite length, estimation of C(t) is not precise for t_i_ → t_N_, since N − i → 0, only few snapshots contribute to the average and ergodicity cannot be assumed any longer due to the sampling problem.

For C(t) to converge, we require it to stay within a certain range of a plateau value S^2^, without large fluctuations, for an extended range of t. We define an error function ε(t) = |C(t) − S^2^| and aim at estimating the longest interval [t_i_;t_j_] such that the mean $\tilde{\varepsilon }_{ij} = \langle \varepsilon \rangle_{ij}$ in this time interval and the corresponding standard deviation σ_ij_ do not exceed 0.05 respectively, as well as $\tilde{\varepsilon }_{{i^{\prime } j^{\prime } }}$ ≤ 0.05 and $\sigma_{{{\text{i}}^{\prime } {\text{j}}^{\prime } }}$ ≤ 0.05 for all sub-intervals [i′;j′] ∈ [i;j]^2^ with i, j = 1…N such that i′ < j′. If 2(i − j) > N, i.e. if C is close to S^2^ for a consecutive time of at least half of its domain definition, we consider C to have converged.

Values of $\tilde{\varepsilon }$ and σ can efficiently be computed using dynamic programming and on-the-fly computation of means and standard deviations in a single pass. However, since we need to compute 0.5 N(N + 1) values of $\tilde{\varepsilon }$ and in our case N is in the range of 2.5 × 10^4^, with ≥10^4^ internal correlation functions to be examined, we decided to divide [1;N] into 10^2^ non-overlapping stretches of equal size, compute the averages of ε on these intervals, and use the 10^2^ averages instead of the 2.5 × 10^4^ original values for further analysis. This has the additional benefit of smoothening the data, without changing the characteristic course of a particular correlation function.

For human human ubiquitin, 56.6 % of the 5,449 correlation functions C_kl_ converge, while 92.4 % of 489 individual protons considered have at least one correlation function which converges; for human FYN tyrosine kinase SH3 domain, 40.5 % of the 2,992 correlation functions converge with 89.6 % of 345 individual protons having at least one converging correlation function; for fibronectin type III domain, 51.6 % correlation functions converge out of 5295 and 93.5 % of 539 protons have at least one converging correlation function; for murine adipocyte lipid binding protein, 50.1 % of 7556 correlation functions converge and 93.1 % of 796 individual protons have at least one converging correlation function.

### Determination of generic order parameters

Distance-dependent order parameters for all pairs of non-exchanging protons with mutual distance less than 5 Å were extracted from the trajectories of four globular proteins, as described above. For the order parameter of proton pair (k, l) S_kl_^2^, a matrix **A** with A_kl_ = S_kl_^2^ was constructed. By applying a conjugant gradient algorithm in MATLAB^®^ (2007a, The MathWorks, Natick, MA), a vector **x** was determined minimizing the *l* ^2^-norm $\left\| {{\mathbf{A}} - {\mathbf{x}}^{\text{T}} {\mathbf{x}}} \right\|.$ This vector holds the *S*-factors S_k_ for all nuclei k, approximating ${\text{S}}_{\text{kl}}^{2} \approx S_{\text{k}} \cdot S_{\text{l}}$ for all pairs of nuclei (k, l), belonging to either the same residue (“intra-residue” dataset) or different residues (“inter-residue” dataset). Protons were classified as belonging to one of 9 different chemical groups, depending on the carbon they are attached to: C-α, CH_3_, CH_2_-β, CH_2_-γ, CH_2_-δ, CH_2_-ε, CH_2_-proline, CH_1_, or aromatic. For each group, the *S*-factors of the protons were averaged over all pairs in the group and over four globular proteins to yield the generic *S*-factor for this group. To restore generic order parameters, protons were assigned generic *S*-factors according to their connectivity, and two *S*-factors were multiplied to retrieve the generic order parameter.

### INPHARMA calculations

As described previously (Orts et al. 2009; Orts et al. 2008), INPHARMA NOEs between the two exchanging ligands for Protein Kinase A (PKA) are computed employing the full relaxation matrix approach (Kalk and Berendsen 1976; Keepers and James 1984; London 1999; Nilges et al. 1991) to account for all possible pathways of spin diffusion, thus allowing for rigorous, quantitative treatment of the NOE transfer. The differential equation

11 $$ \frac{{d{\mathbf{M}}(t)}}{dt} = - ({\mathbf{R}} + {\mathbf{K}})\cdot{\mathbf{M}}(t) $$

is solved for a given NOESY mixing time τ_M_, yielding **M**(*t*), the magnetization matrix at time *t*, as

12 $$ {\mathbf{M}}(\tau_{M} ) = { \exp }( - ({\mathbf{R}} + {\mathbf{K}})\tau_{M} )\cdot{\mathbf{M}}(0) $$

The kinetic matrix **K** represents the chemical exchange according to the kinetic model TL_A_ + L_B_ ↔ L_A_ + TL_B_ with T being the target protein and L_A_ and L_B_ being the respective ligands. The relaxation matrix **R** contains the auto-relaxation rates R_kk_ = ρ_k_ and cross-relaxation rates R_kl_ = σ_kl_ for all nuclei (k, l). The spectral density function used has the form of the first term of Eq. (4), as described in the Theory section.

A more thorough theoretical treatment of INPHARMA can be found elsewhere (Orts et al. 2009).

Equation (11) was solved using a matrix exponential routine of the SciPY library in Python 2.4.3 for mixing times 150, 300, 450, 600 and 750 ms, if not stated differently. Adjustable parameters were set to ω = 800 MHz, proton resonance frequency; τ_c_ = 17 ns, correlation time of the protein; τ_L_ = 100 ps, correlation time of the free ligands; k_AB_ = 3,000 s^−1^ and k_BA_ = 1,000 s^−1^ exchange rates of the respective ligands according to the kinetic model; L_A_ = 450 μM and L_B_ = 150 μM, respective ligand concentrations, and 25 μM (for the NOESY experiments with 450 and 750 ms mixing time) or 30 μM (else) protein concentration, to recapitulate the experimental setup. INPHARMA NOEs were normalized to the intensities of the diagonal peaks of L_A_ in a NOESY spectrum at 150 ms mixing time. Normalized INPHARMA NOEs computed for mixing times 300–750 ms were compared to normalized experimental intensities obtained at the same mixing times, and a simple linear regression was performed to yield the Pearson correlation coefficient and the slope *a* of the regression line y = *a*x.

### Linear regression

Pearson correlation coefficients between samples *x* and *y* and slopes of the best fit line y = *a*x were calculated as R = cov(x,y)/σ_x_σ_y_ and *a* = ∑_i_x_i_y_i_/∑_i_x_i_^2^, respectively, with σ the sample standard deviation.

## Electronic supplementary material

Below is the link to the electronic supplementary material.

Supplementary material 1 (DOCX 540 kb)

## References

[CR1] Berman HM, Westbrook J, Feng Z, Gilliland G, Bhat TN, Weissig H, Shindyalov IN, Bourne PE (2000). The protein data bank. Nucleic Acids Res.

[CR2] Best RB, Rutherford TJ, Freund SM, Clarke J (2004). Hydrophobic core fluidity of homologous protein domains: relation of side-chain dynamics to core composition and packing. Biochemistry.

[CR3] Blommers MJJ, Stark W, Jones CE, Head D, Owen CE, Jahnke W (1999). Transferred cross-correlated relaxation complements transferred NOE: structure of an IL-4R-derived peptide bound to STAT-6. J Am Chem Soc.

[CR46] Brueschweiler R, Roux B, Blackledge M, Griesinger C, Karplus M, Ernst RR (1992). Influence of rapid intramolecular motion on NMR cross-relaxation rates. A molecular dynamics study of antamanide in solution. J Am Chem Soc.

[CR4] Carlomagno T (2005). Ligand-target interactions: what can we learn from NMR?. Annu Rev Biophys Biomol Struct.

[CR5] Carlomagno T, Felli IC, Czech M, Fischer R, Sprinzl M, Griesinger C (1999). Transferred cross-correlated relaxation: application to the determination of sugar pucker in an aminoacylated tRNA-mimetic weakly bound to EF-Tu. J Am Chem Soc.

[CR6] Case DA (2002). Molecular dynamics and NMR spin relaxation in proteins. Acc Chem Res.

[CR7] Constantine KL, Friedrichs MS, Wittekind M, Jamil H, Chu CH, Parker RA, Goldfarb V, Mueller L, Farmer BT (1998). Backbone and side chain dynamics of uncomplexed human adipocyte and muscle fatty acid-binding proteins. Biochemistry.

[CR8] Ernst RR, Bodenhausen G, Wokaun A (1987). Principles of NMR in one and two dimensions.

[CR9] Hamill S, Meekhof A, Clarke J (1998). The effect of boundary selection on the stability and folding of the third fibronectin type III domain from human tenascin. Biochemistry 37(22):8071–8079 10.1021/bi9801659 9609701

[CR10] Hub JS, de Groot BL (2009). Detection of functional modes in protein dynamics. PLoS Comput Biol.

[CR11] Humphrey W, Dalke A, Schulten K (1996) VMD: visual molecular dynamics. J Mol Graph 14(1):33–38, 27–38 10.1016/0263-7855(96)00018-5 8744570

[CR12] Jayalakshmi V, Krishna NR (2002). Complete relaxation and conformational exchange matrix (CORCEMA) analysis of intermolecular saturation transfer effects in reversibly forming ligand–receptor complexes. J Magn Reson.

[CR13] Kalk A, Berendsen HJC (1976). Proton magnetic-relaxation and spin diffusion in proteins. J Magn Reson.

[CR14] Karplus M, Kuriyan J (2005). Molecular dynamics and protein function. Proc Natl Acad Sci U S A.

[CR15] Kay LE, Muhandiram DR, Wolf G, Shoelson SE, Forman-Kay JD (1998). Correlation between binding and dynamics at SH2 domain interfaces. Nat Struct Biol.

[CR16] Keepers JW, James TL (1984). A theoretical-study of distances determination from NMR—two-dimensional nuclear overhauser effect spectra. J Magn Reson.

[CR17] Kohn JE, Afonine PV, Ruscio JZ, Adams PD, Head-Gordon T (2010) Evidence of functional protein dynamics from X-ray crystallographic ensembles. PLoS Comput Biol 6(8):1–5 10.1371/journal.pcbi.1000911 PMC2928775 20865158

[CR18] Leahy DJ, Hendrickson WA, Aukhil I, Erickson HP (1992). Structure of a fibronectin type III domain from tenascin phased by MAD analysis of the selenomethionyl protein. Science.

[CR19] Lee AL, Flynn PF, Wand AJ (1999) J Am Chem Soc 121:2891–2902

[CR20] Lipari G, Szabo A (1982) J Am Chem Soc 104:4546

[CR21] London RE (1999). Theoretical analysis of the inter-ligand overhauser effect: a new approach for mapping structural relationships of macromolecular ligands. J Magn Reson.

[CR22] MacKerell AD (1998). All-atom empirical potential for molecular modeling and dynamics studies of proteins. J Phys Chem B.

[CR23] Markwick PR, Malliavin T, Nilges M (2008). Structural biology by NMR: structure, dynamics, and interactions. PLoS Comput Biol.

[CR24] Mayer M, Meyer B (2001). Group epitope mapping by saturation transfer difference NMR to identify segments of a ligand in direct contact with a protein receptor. J Am Chem Soc.

[CR25] McCoy MA, Wyss DF (2002). Spatial localization of ligand binding sites from electron current density surfaces calculated from NMR chemical shift perturbations. J Am Chem Soc.

[CR26] Ming D, Brueschweiler R (2004). Prediction of methyl-side chain dynamics in proteins. J Biomol NMR.

[CR27] Mittermaier A, Kay LE (2006). New tools provide new insights in NMR studies of protein dynamics. Science.

[CR28] Mittermaier A, Davidson AR, Kay LE (2003). Correlation between 2H NMR side-chain order parameters and sequence conservation in globular proteins. J Am Chem Soc.

[CR29] Ni F (1994). Recent developments in transferred NOE methods. Prog NMR Spectrosc.

[CR30] Nilges M, Habazettl J, Brunger AT, Holak TA (1991). Relaxation matrix refinement of the solution structure of squash trypsin inhibitor. J Mol Biol.

[CR31] Noble ME, Musacchio A, Saraste M, Courtneidge SA, Wierenga RK (1993). Crystal structure of the SH3 domain in human Fyn; comparison of the three-dimensional structures of SH3 domains in tyrosine kinases and spectrin. EMBO J.

[CR32] Orts J, Tuma J, Reese M, Grimm SK, Monecke P, Bartoschek S, Schiffer A, Wendt KU, Griesinger C, Carlomagno T (2008). Crystallography-independent determination of ligand binding modes. Angew Chem Int Ed Engl.

[CR33] Orts J, Griesinger C, Carlomagno T (2009). The INPHARMA technique for pharmacophore mapping: a theoretical guide to the method. J Magn Reson.

[CR34] Orts J, Bartoschek S, Griesinger C, Monecke P, Carlomagno T (2012) An NMR-based scoring function improves the accuracy of binding pose predictions by docking by two orders of magnitude. J Biomol NMR 52(1):23–30 10.1007/s10858-011-9590-5 PMC3266494 22167466

[CR35] Phillips JC, Braun R, Wang W, Gumbart J, Tajkhorshid E, Villa E, Chipot C, Skeel RD, Kale L, Schulten K (2005). Scalable molecular dynamics with NAMD. J Comput Chem.

[CR36] Reese M, Sanchez-Pedregal V, Kubicek K, Meiler J, Blommers M, Griesinger C, Carlomagno T (2007). Structural basis of the activity of the microtubule-stabilizing agent epothilone A studied by NMR spectroscopy in solution. Angew Chem Int Ed Engl.

[CR37] Sanchez-Pedregal VM, Reese M, Meiler J, Blommers MJ, Griesinger C, Carlomagno T (2005). The INPHARMA method: protein-mediated interligand NOEs for pharmacophore mapping. Angew Chem Int Ed Engl.

[CR38] Schneider TR, Brunger AT, Nilges M (1999). Influence of internal dynamics on accuracy of protein NMR structures: derivation of realistic model distance data from a long molecular dynamics trajectory. J Mol Biol.

[CR39] Showalter SA, Brueschweiler R (2007). Validation of molecular dynamics simulations of biomolecules using NMR spin relaxation as benchmarks: an application to the AMBER99SB force field. J Chem Theory Comput.

[CR40] Showalter SA, Johnson E, Rance M, Bruschweiler R (2007). Toward quantitative interpretation of methyl side-chain dynamics from NMR by molecular dynamics simulations. J Am Chem Soc.

[CR41] Trbovic N, Kim B, Friesner RA, Palmer AG (2008). Structural analysis of protein dynamics by MD simulations and NMR spin-relaxation. Proteins.

[CR48] Vijay-Kumar S, Bugg CE, Cook WJ (1987). Structure of ubiquitin refined at 1.8 A resolution. J Mol Biol.

[CR42] Vanommeslaeghe K, Hatcher E, Acharya C, Kundu S, Zhong S, Shim J, Darian E, Guvench O, Lopes P, Vorobyov I, Mackerell AD (2010). CHARMM general force field: a force field for drug-like molecules compatible with the CHARMM all-atom additive biological force fields. J Comput Chem.

[CR43] Vogeli B, Segawa TF, Leitz D, Sobol A, Choutko A, Trzesniak D, van Gunsteren W, Riek R (2009). Exact distances and internal dynamics of perdeuterated ubiquitin from NOE buildups. J Am Chem Soc.

[CR44] Wallach D (1967). J Chem Phys.

[CR45] Word JM, Lovell SC, Richardson JS, Richardson DC (1999). Asparagine and glutamine: using hydrogen atom contacts in the choice of side-chain amide orientation. J Mol Biol.

[CR49] Xu Z, Bernlohr DA, Banaszak LJ (1993). The adipocyte lipid-binding protein at 1.6-A resolution. Crystal structures of the apoprotein and with bound saturated and unsaturated fatty acids. J Biol Chem.

[CR50] Zheng J, Post CB (1993). Protein indirect relaxation effects in exchange-transferred NOESY by a rate-matrix analysis. J Magn Reson.

