# Supplementary material for: The description of protein internal motions aids selection of ligand binding poses by the INPHARMA method
Source: J Biomol NMR. 2012 Sep 22;54(3):245–56. doi: 10.1007/s10858-012-9662-1 (PMC3483107; doi:10.1007/s10858-012-9662-1)
Supplement: Supplementary file 1 — Supplementary material 1 (DOCX 540 kb) [file 10858_2012_9662_MOESM1_ESM.docx]

**Supplementary Material**


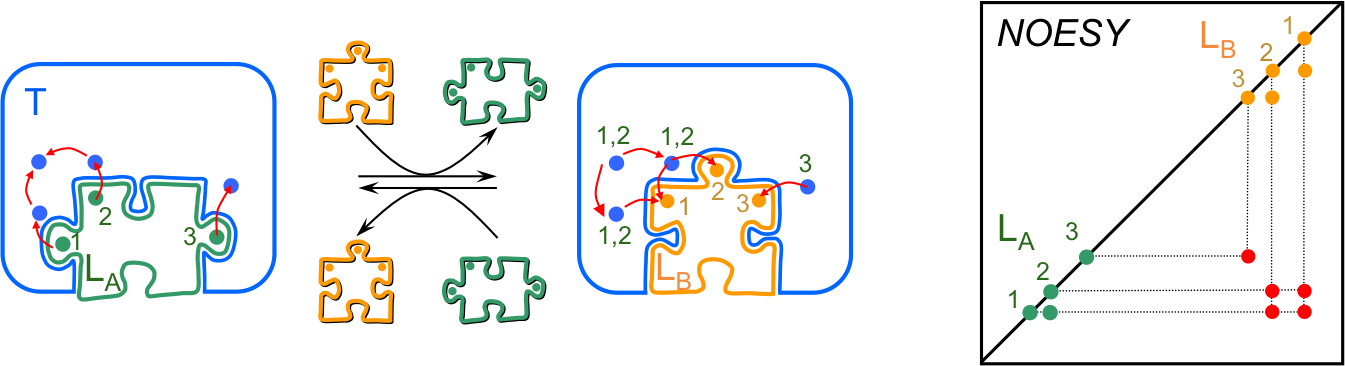


**Supplementary Figure S1.** Origin of inter-ligand signals in an INPHARMA experiment. During the mixing time of a NOESY experiment, L_A_ binds to the receptor T and the magnetization is transferred from the ligand protons 1, 2 and 3 to protons of the binding pocket. Subsequently, L_A_ dissociates and L_B_ binds. Magnetization originating from L_A_ and stored at the protein binding site is transferred to protons of L_B_ 1, 2 and 3 – as this magnetization originates from L_A_, this transfer gives rise to cross-signals between the resonances 1, 2 and 3 of L_A_ and 1, 2 and 3 of L_B_. Thus, INPHARMA NOEs stem from an indirect, spin diffusion mediated transfer, relayed by receptor protons (Orts et al. 2008).


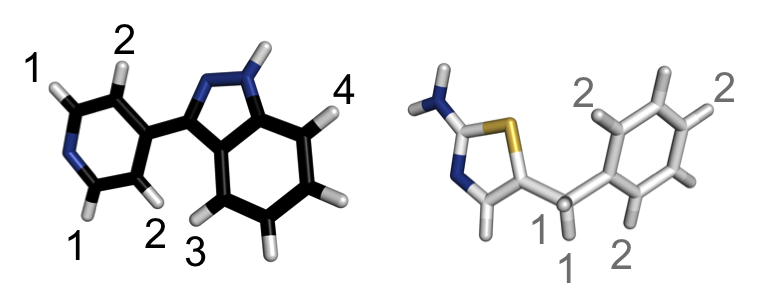


**Supplementary Figure 2.** Protein Kinase A ligands L_A_ (left, black) and L_B_ (right, gray). Atoms are represented as sticks (white: hydrogen, blue: nitrogen, yellow: sulfur, black/gray: carbon). Numbers next to hydrogens indicate distinct groups of overlapped chemical shifts of non-exchanging protons between which INPHARMA-NOEs are observed and can be quantified.

**Supplementary Figure 3.** Expansion of Figure 1 for τ_c_ between 0 and 100 ns.


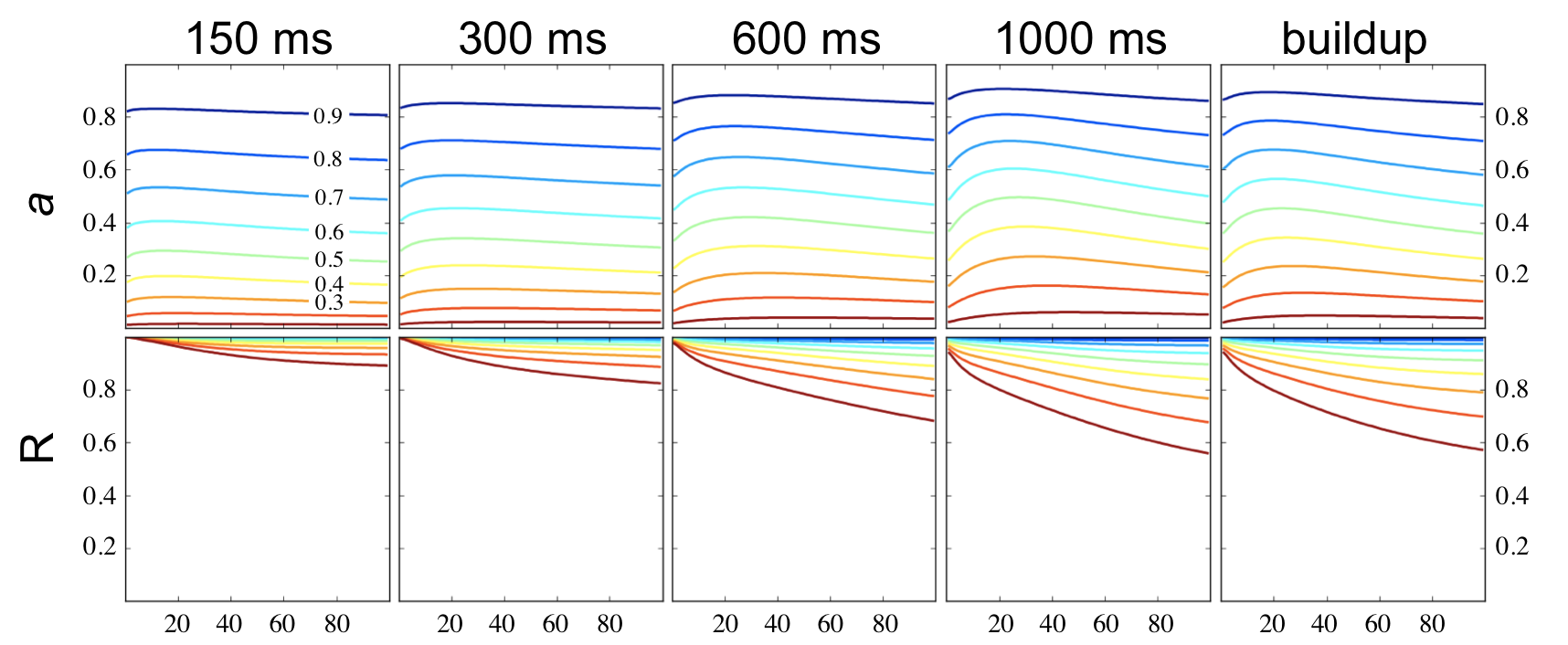


**Table S1.** *S*-factors for “inter-“ and “intra-residue” proton pairs derived from four globular proteins. Both angular and radial fluctuations are contained in these values. N indicates the number of protons used to derive the corresponding S-factor.

|  | Ubiquitin | | | Human FYN tyrosine kinase SH3 domain | | | Fibronectin type III domain | | | Murine adipocyte lipid binding protein | | |
| --- | --- | --- | --- | --- | --- | --- | --- | --- | --- | --- | --- | --- |
|  | Inter  (SD) | Intra  (SD) | N  (inter/intra) | Inter  (SD) | Intra  (SD) | N  (inter/intra) | Inter  (SD) | Intra  (SD) | N (inter/intra) | Inter  (SD) | Intra  (SD) | N  (inter/intra) |
| C-α | **0.97** (0.05) | **0.95** (0.12) | 78/64 | **0.95** (0.08) | **0.89** (0.16) | 62/46 | **0.96** (0.06) | **0.93** (0.13) | 94/75 | **0.96** (0.09) | **0.93** (0.15) | 138/112 |
| CH_3_ | **0.62** (0.13) | **0.55** (0.12) | 146/148 | **0.56** (0.14) | **0.57** (0.12) | 81/73 | **0.62** (0.12) | **0.54** (0.11) | 157/141 | **0.65** (0.12) | **0.57** (0.10) | 227/225 |
| CH_2_-β | **0.88** (0.10) | **0.84** (0.16) | 76/67 | **0.86** (0.12) | **0.81** (0.16) | 63/49 | **0.86** (0.12) | **0.83** (0.18) | 91/73 | **0.87** (0.12) | **0.78** (0.23) | 136/118 |
| CH_2_-γ | **0.79** (0.16) | **0.76** (0.16) | 41/41 | **0.82** (0.19) | **0.74** (0.16) | 15/13 | **0.78** (0.17) | **0.72** (0.21) | 38/36 | **0.76** (0.17) | **0.71** (0.20) | 60/70 |
| CH_2_-δ | **0.69** (0.17) | **0.57** (0.12) | 12/13 | **0.49** (0.05) | **0.67** (0.21) | 3/7 | **0.70** (0.21) | **0.65** (0.20) | 8/10 | **0.70** (0.18) | **0.59** (0.23) | 29/37 |
| CH_2_-ε | **0.43** (0.18) | **0.42** (0.17) | 8/14 | **0.56** (0.00) | **0.38** (0.03) | 1/2 | **0.51** (0.18) | **0.40** (0.18) | 8/6 | **0.40** (0.22) | **0.38** (0.15) | 18/26 |
| CH_2_-_proline_ | **0.84** (0.09) | **0.76** (0.12) | 18/18 | **0.84** (0.11) | **0.77** (0.12) | 12/12 | **0.80** (0.08) | **0.77** (0.13) | 29/30 | **0.83** (0.20) | **0.86** (0.11) | 3/4 |
| CH_1_ | **0.92** (0.14) | **0.75** (0.20) | 24/25 | **0.84** (0.11) | **0.62** (0.19) | 11/12 | **0.92** (0.11) | **0.84** (0.11) | 25/25 | **0.94** (0.09) | **0.84** (0.15) | 38/37 |
| aromatic | **0.76** (0.16) | **0.85** (0.18) | 17/17 | **0.80** (0.12) | **0.89** (0.11) | 36/40 | **0.83** (0.09) | **0.89** (0.17) | 22/24 | **0.74** (0.24) | **0.86** (0.22) | 46/43 |
